# Identifying the domains of context important to implementation science: a study protocol

**DOI:** 10.1186/s13012-015-0325-y

**Published:** 2015-09-28

**Authors:** Janet E. Squires, Ian D. Graham, Alison M. Hutchinson, Susan Michie, Jill J. Francis, Anne Sales, Jamie Brehaut, Janet Curran, Noah Ivers, John Lavis, Stefanie Linklater, Shannon Fenton, Thomas Noseworthy, Jocelyn Vine, Jeremy M. Grimshaw

**Affiliations:** School of Nursing, University of Ottawa, Ottawa, Canada; Clinical Epidemiology Program, Ottawa Hospital Research Institute, Ottawa, Canada; School of Epidemiology, Public Health and Preventive Medicine, University of Ottawa, Ottawa, Canada; School of Nursing and Midwifery, Faculty of Health, Deakin University, Melbourne, Australia; Psychology Department, University College London, London, UK; School of Health Sciences, City University London, London, UK; VA Center for Clinical Management Research, VA Ann Arbor and School of Medicine, University of Michigan, Ann Arbor, MI USA; School of Nursing, Dalhousie University, Halifax, Canada; IWK Health Centre, Halifax, Canada; Department of Family and Community Medicine, Women’s College Hospital, University of Toronto, Toronto, Canada; Department of Clinical Epidemiology & Biostatistics, McMaster University, Hamilton, Canada; Planning, Research and Analysis Branch, Ontario Ministry of Health & Long Term Care, Toronto, Canada; Community Health Sciences, University of Calgary, Calgary, AB Canada; Department of Medicine, University of Ottawa, Ottawa, Canada

## Abstract

**Background:**

There is growing recognition that “context” can and does modify the effects of implementation interventions aimed at increasing healthcare professionals’ use of research evidence in clinical practice. However, conceptual clarity about what exactly comprises “context” is lacking. The purpose of this research program is to develop, refine, and validate a framework that identifies the key domains of context (and their features) that can facilitate or hinder (1) healthcare professionals’ use of evidence in clinical practice and (2) the effectiveness of implementation interventions.

**Methods/design:**

A multi-phased investigation of context using mixed methods will be conducted. The first phase is a concept analysis of context using the Walker and Avant method to distinguish between the defining and irrelevant attributes of context. This phase will result in a preliminary framework for context that identifies its important domains and their features according to the published literature. The second phase is a secondary analysis of qualitative data from 13 studies of interviews with 312 healthcare professionals on the perceived barriers and enablers to their application of research evidence in clinical practice. These data will be analyzed inductively using constant comparative analysis. For the third phase, we will conduct semi-structured interviews with key health system stakeholders and change agents to elicit their knowledge and beliefs about the contextual features that influence the effectiveness of implementation interventions and healthcare professionals’ use of evidence in clinical practice. Results from all three phases will be synthesized using a triangulation protocol to refine the context framework drawn from the concept analysis. The framework will then be assessed for content validity using an iterative Delphi approach with international experts (researchers and health system stakeholders/change agents).

**Discussion:**

This research program will result in a framework that identifies the domains of context and their features that can facilitate or hinder: (1) healthcare professionals’ use of evidence in clinical practice and (2) the effectiveness of implementation interventions. The framework will increase the conceptual clarity of the term “context” for advancing implementation science, improving healthcare professionals’ use of evidence in clinical practice, and providing greater understanding of what interventions are likely to be effective in which contexts.

## Background

Healthcare professionals’ use of research evidence in clinical practice is critical to improving population health and achieving a high-performing health system. Yet, one of the most consistent findings in health services and clinical research is that healthcare professionals’ use of evidence is suboptimal despite increased awareness of and accessibility to research evidence [1–5]. Our understanding of how to improve healthcare professionals’ use of evidence is also incomplete. Implementation science, also known as knowledge translation, is the study of methods to promote the integration of research findings and evidence into healthcare policy and practice. It seeks to understand the behavior of healthcare professionals and other stakeholders as a key variable in the sustainable uptake, adoption, and implementation of evidence-based interventions [6]. In several reviews of implementation studies [7–17], researchers have identified major conceptual and methodological issues facing the field that need to be addressed to improve healthcare professionals’ use of evidence in practice; among them is consistent unexplained variation in intervention effectiveness between trials with a possible explanation being the influence of context. To advance the field, we therefore need to begin considering and measuring context; this first requires a clear conceptualization of the key domains (and their features) of context that are likely to influence variation in the effectiveness of implementation interventions.

### Defining context

While context is broadly known as the physical and social environment, the term is used differently by different authors. More specifically, there is little agreement about what domains, measures, and features of context are important to healthcare professionals’ use of evidence. For example, Øvretveit [18] defines context broadly as all factors that are not part of the intervention. May et al. [19] adopt a more specific definition as follows: “the physical, organizational, institutional, and legislative structures that enable and constrain, and resource and realize, people and procedures”. French et al. [20] define context as “the organizational environment of healthcare, composed of physical, social, political and economic influences on the practical reasoning and choices of practitioners about how clinical issues are addressed” (p. 174) while Rycroft-Malone [21] defines it as “the environment or setting in which the proposed change is to be implemented” (p.299). Further, GW Allport’s [22] seminal definition from social psychology highlights the effect of the “real, imagined or implied presence of others” on behavior, implying that a social context exists that is much broader than the immediately observable features of the environment.

Context will vary by setting; however, a core set of domains of context that are important to all or most healthcare professionals’ use of evidence in clinical practice is likely to exist. While each domain may be more or less important in different settings, each should, at minimum, be assessed prior to designing and delivering implementation interventions.

### Context and implementation interventions

Empirical evidence supporting the central role of context to implementation interventions has emerged in studies where intervention effectiveness varied by context. For example, Hogg et al. [23, 24] conducted two trials on the effects of practice facilitation in improving preventive care delivery and found benefits in capitation-based practices but not fee-for-service practices, illustrating that context matters to intervention effectiveness. Similar findings were reported in a recent review of point-of-care computer reminders; interventions that targeted inpatient settings had larger improvements in processes of care than those in outpatient settings [25]. A study commissioned by the UK’s Health Foundation identified essential contextual characteristics for successful implementation of a program (*Keystone*, conducted in Michigan, USA) and then explored what happened when a program it inspired (*Matching Michigan*) was launched. They reported that application of the program’s technical practices were generally very good, but implementation of the broader set of factors shown to be relevant to success in the original program was highly variable and depended on the national, local, and internal context [26]. These examples illustrate the need to consider scope and dimensions of context when designing implementation interventions in healthcare when interpreting trial findings and when considering the limits of generalizability (external validity) of trial findings.

### Frameworks for context

While several implementation frameworks acknowledge the importance of context, authors provide little detail on what they consider to be the key domains of context. Rogers’ *Diffusion of Innovations* [27] is the most frequently used theory in studies of implementation in clinical practice [28]. It describes organizational innovativeness as being related to a variety of contextual features such as: leadership, internal organizational structure (centralization, complexity, formalization, interconnectedness, organizational slack), and external characteristics of the organization [27]. The *Promoting Action on Research in Health Services* (*PARiHS*) framework was developed to explain healthcare professionals’ use of evidence in clinical practice. It is hypothesized to be a function of (i) the sources of evidence used to support practice change, (ii) the context (defined as three domains—leadership, culture, and feedback) where practice change occurs, and (iii) methods used to facilitate practice change [29, 30]. The *Knowledge to Action Cycle* [31] also highlights the importance of context to successful implementation in clinical practice and its assessment to inform the design and delivery of implementation interventions. Included in the action part of the cycle are processes related to context that are needed to translate research in healthcare settings, namely: applying knowledge (research) to the local context and the assessment of barriers and supports to knowledge (research) use (which includes consideration of the local context). Damschroder et al. [32] describe their *Consolidated Framework for Implementation Research* (*CFIR*), which recognizes the multiple levels at which contextual influences on behavior change operate and allows for interaction between contextual factors insofar as they influence clinical practice. The framework includes 8 concepts related to the intervention itself, 4 related to the outer context (such as the patient and resources), 12 related to the inner setting or context (such as culture and leadership), 5 related to the individual, and 8 related to processes (such as planning and reflection). This framework reflects the organizational and policy literature, where context is examined from the perspective of levels. In the field of health psychology, there is the Theoretical Domains Framework [33, 34]. This framework focuses on individuals’ perceptions of the determinants of their behavior. It consists of 14 domains derived from 128 explanatory constructs from 33 health and social psychology theories. The framework explicitly recognizes context in 2 of the 14 domains (social influences and environmental context). Details, however, on the specific features of context that would fall into these domains have not been investigated. In the organizational literature, three levels of context are commonly proposed: macro in which market-type forces are at play (e.g., growth in strategic management), meso in which organizational characteristics are an influence, and micro in which activities in the clinical setting provide a contextual influence [35]. The organizational literature also suggests that multiple levels of context create a layered set of influences and require examination of influence at each level.

Despite the abundance of frameworks that identify context as an influence on implementation, several unanswered questions about context remain. *First*, while several implementation frameworks include context, no one framework is sufficiently inclusive or comprehensive about what comprises context. *Secondly*, existing frameworks are often inconsistent with one another regarding how they define context and what they consider to be the important domains of context. As a result, there is little direction on which elements of context need to be assessed prior to designing an implementation intervention to improve healthcare professionals’ use of evidence in clinical practice. *Thirdly*, knowledge users (e.g., healthcare decision makers, change agents) have not always been engaged in developing the existing frameworks, meaning their knowledge on what domains of context are important to evidence use by healthcare professionals and to the success of implementation interventions is lacking in these frameworks. This may limit knowledge users’ acceptance and thus use of the frameworks to assess context to inform their implementation efforts. *Fourthly*, the existing frameworks were not subject to international content validation with expert-user groups, which is critical to ensuring that the framework is acceptable and useful to its users [36].

Increasing clarity about the concept of context will lay the basis for increasing understanding of context for advancing implementation science, designing and delivering effective implementation interventions, and reducing variation in the effectiveness of these interventions. Because of the different levels and multiple features of context, which may indicate different domains of context, we believe it is important to construct a framework that describes context both fully and precisely, supporting development of the science of implementation research. Therefore, the purpose of the research program described in this manuscript is to develop, refine, and validate a comprehensive framework of context that identifies the domains of context (and their features) that can facilitate or hinder (1) healthcare professionals’ use of evidence in clinical practice and (2) the effectiveness of implementation interventions.

## Methods

### Design and objectives

This project is a multi-phased investigation of context using mixed methods.

The specific objectives of this project are:

1. To conduct a concept analysis of context as it is described in the international literature in order to develop a preliminary framework of domains of context (and their features) important to healthcare professionals’ use of evidence in clinical practice (Context study 1)
2. To conduct a secondary analysis of qualitative data collected from healthcare professionals internationally on the perceived contextual barriers and enablers to their use of evidence in clinical practice (Context study 2)
3. To conduct interviews with a variety of international health system stakeholders and change agents to elicit their knowledge and beliefs about contextual features that influence the effectiveness of implementation interventions and healthcare professionals’ use of evidence in clinical practice (Context study 3)
4. To synthesize data from the three context studies in order to refine the framework of context drawn from the concept analysis—Context study 1 (Data Triangulation)
5. To assess the resulting context framework for content validity through an iterative process involving expert researchers and health system stakeholders and change agents (Delphi Study).

### Context study 1: concept analysis

A protocol describing our methods for the concept analysis is published elsewhere [37]. We are using a modified Walker and Avant method of concept analysis [38] comprising six systematic steps: (1) concept selection, (2) statement of the aims and purpose of the concept analysis, (3) identification of uses of context, (4) determination of defining attributes of context, (5) identification/construction of different cases of context (i.e., related cases, borderline cases, contrary cases, illegitimate cases, and invented cases), and (6) definition of empirical referents. For further details, see Squires et al. [37].

### Context study 2: secondary analysis of interviews with healthcare professionals

A secondary analysis of qualitative (semi-structured interview) data investigating healthcare professionals’ perceived barriers and enablers to their use of evidence in clinical practice will be conducted. While secondary analysis of quantitative (e.g., survey) data is increasingly undertaken, data sharing among qualitative researchers is less common. According to Cotri and Thompson [39, 40] and Heaton [41, 42], re-use of qualitative datasets is infrequent and comprises untapped “resources”. For this phase of our research program, a unique collection of qualitative data investigating healthcare professionals’ perceived barriers and enablers to their use of evidence in clinical practice has been compiled. The original datasets were collected using similar methods across different countries, healthcare professionals, settings, and behaviors, providing richness not available in any single dataset. The data were collected using semi-structured interview guides informed by the Theoretical Domains Framework [33, 34] (see Background). Interview questions were broad and open-ended, allowing participants to spontaneously identify instances of context that act as barriers and/ or enablers to their use of evidence in clinical practice. The target behaviors in each of these studies were specified with a high level of granularity (see Table 1), but all involved the application of evidence from clinical research and/or clinical guidelines As a result, these data provide a rich array of contextual features.

**Table 1:** Healthcare Professional Datasets (*N* = 13 Datasets)

	Sample				TACT			
Dataset	*N*	Professional group	Country	Data collection dates	Target	Action	Context	Time
Red blood cell transfusion-1	12	Intensivists	Canada	April–October 2008	Patients	Watching and waiting vs. infusing red blood cells	Intensive care units	When patient has borderline hemoglobin
Red blood cell transfusion-2	12	Orthopedic surgeons	Canada	September–July 2009	Patients	Watching and waiting vs. infusing red blood cells	General surgery wards	When patient has borderline hemoglobin
Pre-operative assessment	11	Anesthesiologists	Canada	September–October 2009	Patients	Completing an assessment without a routine electro-cardiography	Pre-assessment units	During pre-operative assessments
	5	Surgeons						
Smoking cessation	10	Family physicians	Canada	March–October 2009	Patients	Adherence to a guideline for smoking cessation	Primary care	During patient visit
Fetal monitoring	12	Labor & delivery nurses	Canada	April–May 2010	Patients	Intermittent auscultation for fetal surveillance	Birthing units	During labor
Adult computerized tomography head rule	8	Emergency room physicians	Canada	March–June 2010	Patients	Using an adult computerized tomography head rule	Adult emergency room	During emergency room visit for a head injury
Child computerized tomography head rule	31	Physicians	Canada	January–July 2011	Patients	Using a child computerized tomography head rule	Pediatric emergency room	During emergency room visit for a head injury
	11	Nurses						
Bone mineral density screening	12	Family physicians	Canada	September–November 2012	Patients ≥50 years	Order a bone mineral density screen	Physician’s office	At next available appointment when find out about Fragility Fracture
Hand hygiene	22	Physicians	Canada	September 2012–Feb 2013	Patients	Hand hygiene	Medical and surgical wards	Before initial contact, after contact, before aseptic procedures, and after bodily fluid exposure
	20	Residents						
	4	Infection Control Specialists						
Donation after cardio-circulatory death	24	Intensivists	Canada	October–July 2014	Patients	Donation after cardio-circulatory death	Hospitals that perform organ donation	At circulatory death
	16	ICU nurses						
	15	Organ Donor Coordinators						
Fit: feedback intervention trial	12	Doctors	UK	February 2006	Patients	Hand hygiene	Intensive care units and elderly medical units	While in the hospital
	32	Nurses						
Preconception care guidelines	22	Physicians	Australia	October–November 2007	Patients	Adherence to guidelines for preconception care	Primary care (general practitioner office)	During patient visit
Lower back imaging	21	Chiropractors	Canada and USA	February–July 2010	Patients	Managing back pain without an x-ray	Private clinics	During patient visit

#### The sample and data

The sample consists of 13 individual datasets including interviews from 312 healthcare professionals in four countries: Canada, USA, UK, and Australia. A variety of healthcare professionals are included, for example, different specialties of physicians (intensivists, orthopedic surgeons, general surgeons, anesthesiologists, family physicians, emergency physicians, infectious diseases physicians), and nurses (labor and delivery, emergency, critical care, infection control), trainee doctors (residents), chiropractors, and organ donor coordinators. A variety of clinical settings are also included: different types of hospital units (intensive care, medical, surgical, pre-operative, birthing, adult and pediatric emergency rooms) and primary care settings (private clinics, physician offices). The 13 datasets are summarized in Table 1 using the TACT principle as follows: Target (population the behavior is performed toward), Action (act that you plan to intervene upon), Context (the clinical setting), and Time (timeframe when the action occurs) [43].

#### Data analysis

Data analysis will be managed using NVIVO software [44]. All transcripts in a single dataset will be analyzed before proceeding to the next dataset. Data will be analyzed inductively, following constant comparative analysis [45, 46]. First, two team members will independently read four transcripts from dataset number 1 to determine a coding scheme comprising codes, definitions of codes, and examples of utterances that align with each code. Two team members will then independently analyze the remaining transcripts using the coding scheme. The coding scheme will be modified as needed throughout the analytic process. Analysis will occur in three steps: selection of utterances, coding, and categorizing. Each transcript will first be read and key ideas (i.e., utterances) that reflect context highlighted. These context utterances will then be assigned a “code”. Codes will be operationally defined in order to be consistently applied throughout the data. Codes will then be placed into broad categories, which will become our major units of analysis. Comparisons between multiple categories will be carried out in order to locate similarities and differences between them. Each category of context identified in the transcripts will be given a label, definition, and guideline for identification. Individuals responsible for analysis will meet biweekly to compare their interpretations and jointly select a label that best represents each category and determine a definition and guideline for its identification, revising the coding scheme as needed. Coder reliability will be assessed using the Miles and Huberman [47] approach: Coder reliability = number of agreements/(total number of agreements + disagreements). In instances of disagreement, the rationale behind the coding will be discussed to reach consensus. When agreement is good (≥70 %) [47], the labels, definitions and guidelines for identification by the two coders will be discussed and synthesized to provide the clearest articulation. Reasons for disagreements will be discussed and consensus sought. When consensus is reached, the label, definition, and/or guidelines for identification will be improved and inter-rater agreement reassessed using two new coders.

### Context study 3: semi-structured interviews with health system stakeholders and change agents

#### Sample

The sample is health system stakeholders and change agents who are responsible for the design and implementation of implementation interventions, programs, and change processes that are focused on improving healthcare professionals’ use of evidence in clinical practice. Participants will be purposefully selected from the four countries represented by our team: Canada, USA, England, and Australia. These countries have different health systems; by sampling across these systems, we will capture variation in macro contextual factors. We will purposefully sample from each country to ensure that we interview a variety of participants, for example within (1) public health systems (where the government predominantly covers costs), (2) private health systems (where individuals predominantly cover costs through private insurance premiums), and (3) managed care health system (where there are institutional arrangements that put administrators and designated “gatekeepers” in charge of guiding patients through a healthcare network to manage costs). Because there will also be different health systems within public, private, and managed care programs by country, we anticipate recruiting 12–20 individuals across settings (e.g., inpatient, outpatient, long-term care, home care) working in each of (1) public, (2) private, and (3) managed care systems, for a total of 36–60 interviews for this phase of our research program.

#### Data collection and analysis

Semi-structured key informant interviews will be conducted using an interview guide designed to elicit participants’ knowledge and beliefs about context (what it is and the contextual domains and their features that are perceived to influence implementation interventions and healthcare professionals’ use of evidence in clinical practice). Interviews are expected to last 45 min, be conducted by telephone, and will be digitally recorded (with consent). To monitor the progress of the interviews, permit follow-up of issues that may emerge from the data, and allow us to assess whether we have reached data saturation, interviewing, transcription, and analysis will proceed concurrently. The digital recordings will be transcribed verbatim and verified by the interviewer prior to analysis. Inductive data analysis using constant comparative analysis, as described under Context study 2, will be used.

### Data triangulation

The findings from the three context studies described above (the concept analysis, the secondary analysis of healthcare professional interviews, and the interviews with health system stakeholders and change agents) will be triangulated in order to refine and increase confidence about the comprehensiveness of the preliminary context framework derived from the concept analysis. We will follow a 6-step triangulation process as outlined by Farmer and colleagues [48] and as summarized in Table 2. Two groups of team members will independently undertake the triangulation process and compare their results. Data triangulation will result in an extensive framework of context domains (and their features) important to healthcare professionals’ use of research evidence in clinical practice and the effectiveness of implementation interventions. The framework will represent a shared understanding of context across researchers in the published literature internationally (Context study 1), healthcare professionals (Context study 2), and health system stakeholders and change agents (Context study 3).

**Table 2:** The triangulation process [48]

Step	Description
1. Sorting	• The findings from each context study will be sorted and separated into three files (1 file/context study)
	• The contents of each file will then be reviewed to identify its key categories
2. Convergence coding	• A convergence-coding matrix will be created to compare the three files with respect to:
	° the meaning and interpretation of categories
	° the frequency and prominence of categories
	° specific examples supporting or explaining a particular category
	• A convergence-coding scheme will then be applied:
	• *Agreement* (full agreement between the sets of results on both elements of comparison, e.g., meaning and prominence are the same)
	• *Partial agreement* (agreement on one but not both components, e.g. meaning or prominence of categories is the same)
	• *Silence* (one set of results covers the category or example while another set of results is silent on the category or example)
	• *Dissonance* (disagreement between the sets of results on both elements of comparison, e.g., meaning and prominence are different; and specific examples provided are different)
	• The purpose of this coding scheme is to:
	° determine convergence between the three sets of results on the identity and prominence of the categories presented
	° determine convergence of the coverage and specific examples provided in relation to each category
3. Convergence assessment	• All compared segments will be reviewed to provide a global assessment of the level of convergence
	• When and where team members have different perspectives on convergence or dissonance will be documented
4. Completeness comparison	• The nature and scope of the context domains (and their features) will be compared for each inquiry to enhance the completeness of our united set of findings and identify key differences in scope/coverage
	• In this step, the aim is to broaden the range of findings to ensure completeness in perspective and how a category is characterized
	• To obtain a holistic view of the data, categories will now also be synthesized into broader themes through consensus
5. Team member comparison and 6. feedback	Steps 5 and 6 will occur concurrently
	• The assessments of convergence/dissonance and completeness of the united set of findings among the team will be compared to:
	° clarify interpretations of the findings and
	° reach consensus
	• At a face-to-face team meeting, a 1-day discussion will be held where findings of the triangulation will be provided to the full research team for feedback and discussion
	• In instances of disagreement, the rationale behind the assigned categories
	• and themes will be discussed to come to consensus. Level of agreement between the team should meet or exceed 70
	• Any changes proposed by the team (where they can be accommodated by overall findings) into final data interpretation will be incorporated

### Delphi study: assessment of the context framework for content validity

The final phase of our research program is to assess the content validity of the refined context framework resulting from the data triangulation phase. A modified Delphi approach [49–51] will be used. International experts will be asked to quantitatively rate their agreement with the domains (and their features) in the framework given the data and the processes used to derive the framework. The Delphi approach was selected because it is anonymous and thus will allow participants from different backgrounds to participate without imposing a hierarchy. It has previously been used for similar purposes, for example, to identify innovation determinants in healthcare [52] and to confirm research priorities in different health settings [53–56].

#### Sampling

Participants will be selected from the international research and healthcare delivery community and will include (1) researchers from a variety of disciplines (e.g., health services, implementation science, quality improvement, public health, social science, behavioral science, organizational science) that study context and implementation in healthcare and (2) health system stakeholders and change agents who are responsible for the design and delivery of interventions, programs, and change processes focused on increasing evidence-use by healthcare professionals in clinical practice. There is a broad range of estimates of suitable sizes for a Delphi panel. Lumley et al. [57], however, showed that any sample larger than *n* = 65 can be treated as normally distributed, allowing the use of large sample estimators with more robust properties in Delphi analyses. Therefore, 70 participants will be recruited. Assuming a 50 % response rate [58], 140 experts will be invited to participate.

#### Data collection

Participants in the Delphi process will complete 2–3 email questionnaires estimated to take 20–30 min to complete *Round 1*. Participants will be provided with (1) a description of the process used to develop the context framework, (2) a data triangulation table that summarizes the framework domains (and their features) of context with supporting evidence from the three context studies conducted, and (3) a questionnaire that asks the experts to rate their strength of agreement (on a 9-point scale [59, 60]) with the domains (and their features) in the context framework given the data provided. There will also be open-ended fields to allow participants to provide comments on each domain and on any domains/features they interpret as missing, *Round 2*. Quantitative data (frequency distributions and measures of spread) from Round 1 will be fed back by email to the experts who will be asked to confirm or revise their initial views, *Round 3*. Round 2 will be repeated if needed. A maximum of three rounds will be conducted to limit participant burden.

#### Data analysis

All participants will be treated and analyzed as a single group of experts. The initial ratings of agreement from Round 1 will be summarized as frequency distributions together with measures of dispersion (interquartile ranges) and central tendency (mean, median). In line with previous Delphi studies, participants will be considered as in “disagreement” if 30 % or more of the ratings are in the lower third (ratings 1–3), and 30 % or more of the ratings are in the upper third (ratings 7–9) [60]. Context domains (and features) with an overall rating of 7 to 9 (without disagreement) will be judged as appropriately included in the framework [60]. Agreement ratings from Round 2 will also be summarized quantitatively (as outlined above) and presented back to the participants. A third round will be conducted only if necessary using the same procedure. The Delphi process will be ceased when agreement is established on inclusion of 70 % of the context domains (and their features), defined by a Wald statistic of <0.7, or there is no change in participant scores between two consecutive rounds, defined as a change in the mean score across all participants of >1 scale point for any individual item. Following completion of the Delphi process, any revisions necessary will be made to the context framework.

## Discussion

This paper presents the protocol of a multi-phased research program that takes up the challenge to develop, refine, and validate a comprehensive framework of context that identifies the key domains (and their features) of context that facilitate and hinder (1) healthcare professionals’ use of evidence in clinical practice and (2) the effectiveness of implementation interventions. The framework will represent a *shared understanding* of context that is needed to advance the science of implementation. The framework will be useful to both researchers and knowledge users (healthcare decision makers, implementers/change agents). Researchers and knowledge users may be able to use the framework to guide a priori assessments of context (to assist in the design and delivery of their implementation interventions) as well as a posteriori assessments of context (to aid the interpretation of the effects of implementation interventions which can then inform the design and delivery of subsequent implementation trials). Researchers and knowledge users may also be able to use the framework to pragmatically guide their implementation efforts by identifying important features of context that are important to consider when choosing, designing, and delivering implementation interventions, and to help assess the transferability of effective implementation interventions from other contexts to theirs by identifying contextual features that may influence effective implementation.
